# EukRef-excavates: seven curated SSU ribosomal RNA gene databases

**DOI:** 10.1093/database/baaa080

**Published:** 2020-11-20

**Authors:** Martin Kolisko, Olga Flegontova, Anna Karnkowska, Gordon Lax, Julia M Maritz, Tomáš Pánek, Petr Táborský, Jane M Carlton, Ivan Čepička, Aleš Horák, Julius Lukeš, Alastair G B Simpson, Vera Tai

**Affiliations:** Biology Centre, Institute of Parasitology, Czech Academy of Sciences, 370 05 České Budeějovice (Budweis), Czech Republic; Faculty of Science, University of South Bohemia, 370 05 České Budeějovice (Budweis), Czech Republic; Biology Centre, Institute of Parasitology, Czech Academy of Sciences, 370 05 České Budeějovice (Budweis), Czech Republic; Institute of Evolutionary Biology, Faculty of Biology, Biological and Chemical Research Centre, University of Warsaw, 02-089 Warsaw, Poland; Department of Parasitology, BIOCEV, Faculty of Science, Charles University, 128 43 Vestec, Czech Republic; Department of Biology and Centre of Comparative Genomics and Evolutionary Bioinformatics, Dalhousie University, Halifax, NS B3H 4R2, Canada; Department of Biology, Center for Genomics and Systems Biology, New York University, New York, NY 10003, USA; Department of Zoology, Charles University, 128 00 Prague, Czech Republic; Biology Centre, Institute of Parasitology, Czech Academy of Sciences, 370 05 České Budeějovice (Budweis), Czech Republic; Department of Biology, Center for Genomics and Systems Biology, New York University, New York, NY 10003, USA; Department of Zoology, Charles University, 128 00 Prague, Czech Republic; Biology Centre, Institute of Parasitology, Czech Academy of Sciences, 370 05 České Budeějovice (Budweis), Czech Republic; Faculty of Science, University of South Bohemia, 370 05 České Budeějovice (Budweis), Czech Republic; Biology Centre, Institute of Parasitology, Czech Academy of Sciences, 370 05 České Budeějovice (Budweis), Czech Republic; Faculty of Science, University of South Bohemia, 370 05 České Budeějovice (Budweis), Czech Republic; Department of Biology and Centre of Comparative Genomics and Evolutionary Bioinformatics, Dalhousie University, Halifax, NS B3H 4R2, Canada; Department of Biology, University of Western Ontario, London, ON N6A 5B7, Canada

## Abstract

The small subunit ribosomal RNA (SSU rRNA) gene is a widely used molecular marker to study the diversity of life. Sequencing of SSU rRNA gene amplicons has become a standard approach for the investigation of the ecology and diversity of microbes. However, a well-curated database is necessary for correct classification of these data. While available for many groups of Bacteria and Archaea, such reference databases are absent for most eukaryotes. The primary goal of the EukRef project (eukref.org) is to close this gap and generate well-curated reference databases for major groups of eukaryotes, especially protists. Here we present a set of EukRef-curated databases for the excavate protists—a large assemblage that includes numerous taxa with divergent SSU rRNA gene sequences, which are prone to misclassification. We identified 6121 sequences, 625 of which were obtained from cultures, 3053 from cell isolations or enrichments and 2419 from environmental samples. We have corrected the classification for the majority of these curated sequences. The resulting publicly available databases will provide phylogenetically based standards for the improved identification of excavates in ecological and microbiome studies, as well as resources to classify new discoveries in excavate diversity.

## Introduction

The small subunit ribosomal RNA (SSU rRNA) gene is the most widely used molecular taxonomic marker for microorganisms (16S rRNA for Bacteria and Archaea, and 18S rRNA for Eukaryota). This is primarily because of its universality, sequence variation and data availability from organisms across the tree of life. Indeed, the global SSU rRNA dataset encompasses a larger breadth of diversity than any other reference sequence resource. The identification and classification of a particular organism of interest can be determined by comparing full-length SSU rRNA gene sequences, or even just shorter variable regions, with a taxonomically annotated sequence database. Sequencing short amplified fragments of the SSU rRNA gene from environmental DNA and RNA (‘amplicon sequencing’) has become a crucial standard tool for studying the ecology of microbial organisms, since it provides a culture-independent method to catalogue and assess the diversity of microorganisms from environmental samples (1).

Use of the SSU rRNA as a taxonomic marker gene requires an accurate assignment of sequence data to existing species and manual curation of available metadata. Correct assignment of short-read sequences to existing taxa is thus utterly dependent on the quality of the reference database. Indeed, microbial ecology requires well-curated, community-based reference datasets for every significant group of eukaryotes, allowing researchers to analyze the diversity of a given microbial community properly. Such databases are generally not readily available for microbial eukaryotes, which significantly hinders studies based on environmental DNA. A high-quality reference database can be generated through collection of all relevant SSU rRNA gene sequences and their careful curation by experts on the diversity of each particular lineage. In this article, we present a reference database for the excavate protists, assembled using the EukRef protocol (2) and curated by experts on the major taxa within this assemblage.

The excavates represent a large slice of microbial eukar-yote diversity and are often considered to represent a supergroup—Excavata. There are two main groups: Metamonada (with three subgroups—Fornicata, Parabasalia and Preaxostyla) and Discoba (with four subgroups—Euglenozoa, Heterolobosea, Jakobida and *Tsukubamonas*) (3–5). One other small taxon, Malawimonadida, is historically and often still considered an excavate group (6), but contains just two described species and will not be treated here. Historically, the monophyly of Excavata was inferred using ultrastructural features combined with partial molecular phylogenetic evidence (3, 7). Presently, it is unclear whether excavates are monophyletic; some recent phylogenomic analyses infer that Metamonada and Discoba form a clade (but are not specifically related to Malawimonadida), while others place them as separate deep-branching lineages amongst eukaryotes, sometimes with Metamonada as sister to Malawimonadida (6, 8).

Excavates are ecologically incredibly diverse microbial eukaryotes, including heterotrophs and autotrophs as well as commensals and parasites of diverse hosts (7, 9–11). They also commonly inhabit extreme environments, including hypersaline and anoxic habitats (12–14). Many excavates are of great medical and veterinary importance and human pathogens are found in the genera *Giardia* (responsible for diarrhea), *Trichomonas* (causing urogenital disease), *Trypanosoma* and *Leishmania* (causative agents of African sleeping sickness, Chagas disease, and various leishmaniases), and *Naegleria fowleri*, which is an opportunistic pathogen that causes rare but invariably deadly primary amoebic encephalitis. *Tritrichomonas foetus* is a severe pathogen of cattle that causes abortion of fetuses, and *Histomonas meleagridis* causes lethal systemic infections of poultry (15). In addition to these medically important species, many excavates are essential components of various microbial ecosystems. A wide diversity of Parabasalia and Oxymonadida inhabit the hindgut of wood-eating insects, mainly termites, where they digest cellulose as indispensable symbionts (16). Diplonemea, a previously poorly known group of Euglenozoa, were recently inferred to be the most diverse eukaryotes in the marine plankton (17, 18). Finally, the Heterolobosea dominate the known diversity of smaller protists in hypersaline habitats (19).

SILVA and PR2 are the two main public rRNA databases that include eukaryotes. The PR2 database specifically includes an updated, standardized eukaryotic taxonomy of eight ranks (a set number of ranks being necessary for computational classification) (20, 21). However, neither SILVA or PR2 taxonomies are curated by experts on each particular group, and many unassigned sequences might be assigned more complete classifications based on phylogenetic trees and expert curation. To improve upon these databases, the EukRef initiative is in the process of building databases of SSU rRNA gene sequences for all major eukaryotic groups, with taxonomic assignments curated by experts and confirmed by phylogenetic analyses, and with sequences appended with pertinent metadata, such as environmental and/or geographic origin, and biotic relationships (2; eukref.org). EukRef databases use a widely accepted classification based on morphology and phylogeny (5) that allows a varying number of hierarchical levels for different eukaryotic groups, as well as standardized informal names for clades composed only of environmental sequences that lack a formal taxonomic name. These classifications also take into account the limitations of SSU rRNA gene sequences in discriminating recent evolutionary divergences (i.e. high sequence similarity between species) by assigning taxon names to taxonomic ranks only if they can be distinguished in SSU rRNA gene phylogenies, at the species level or otherwise. As such, EukRef databases, which only comprise SSU rRNA sequences longer than 500 bp, are intended to serve as a gold-standard reference for the assignment and classification of eukaryotic rRNA sequences, especially short-read sequences.

For the Excavata, 6121 SSU rRNA sequences have been curated, all of which required improvements or corrections from the classifications in GenBank, particularly at the highest taxonomic ranks. The presented curated databases are publicly available at the official EukRef repository (https://github.com/eukref/curation) and will be also assimilated into future PR2 database releases (https://github.com/pr2database/pr2database). We also summarize current knowledge of species diversity of the analyzed lineages and their SSU rRNA-based phylogeny, as well as their geographic distributions. Going forward, these databases should aid in choosing the most promising sampling sites to isolate and further characterize as-yet unknown or uncultured lineages and in designing projects focused on particular groups of excavates in habitats where they are the most abundant and diverse.

## Results and discussion

### SSU rRNA global phylogeny

**Figure 1.**
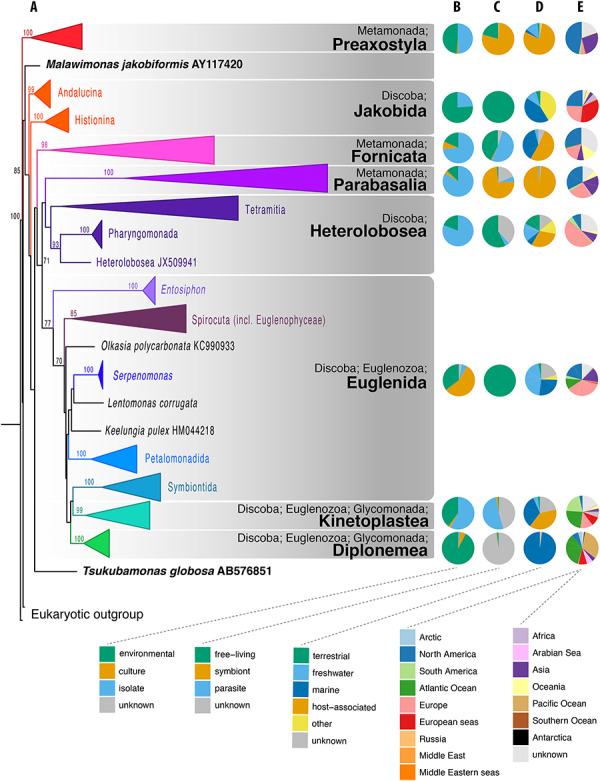
Phylogenetic and compositional overview of the Excavata EukRef databases. (A) Maximum likelihood phylogenetic tree of SSU sequences in the Excavata database. Monophyletic clusters corresponding to deep-level taxa within the Excavata were collapsed at their common ancestral nodes when strongly supported by bootstrapping. The tree was constructed using a GTRCAT nucleotide substitution model. Bootstrap values are shown at nodes with at least 70% support. (B–E) Pie charts showing the proportion of sequences for metadata categories for each Excavata database. (B) Source of the organism from which the SSU sequence was derived. ‘Environmental’ indicates sequences obtained from the DNA of bulk environmental samples. ‘Culture’ indicates sequences obtained from organisms grown in culture and in culture collections. ‘Isolate’ indicates sequences obtained from organisms isolated from the environment, either as single cells or in enrichments, but not from established cultures (C) Biotic relationship of the organism. Symbiont consists of organisms with mutualist or commensal relationships, (D) Environment from which the organism was sampled, (E) Geographical location of sampled environment.

We have constructed a phylogenetic tree of excavates based on all available SSU rRNA sequence diversity (Figure 1A, NCBI NR database as of July 2018). This single-gene tree is poorly resolved (most deep internal nodes receive lower than 80% bootstrap support), moreover, neither Metamonada nor Discoba form a clade. Excavates remain one of the most challenging groups for phylogenetic analyses because both Metamonada and Discoba include many extremely divergent SSU rRNA gene sequences as well as slow-evolving lineages resulting in shorter branches (4, 6). Their wide recognition as clades, however, is due to multigene and phylogenomic analyses, and not SSU rRNA trees (4, 6, 8). It is therefore not surprising that a phylogenetic tree based solely on the SSU rRNA gene, even with the best available taxon sampling, still poorly resolves the phylogeny of the excavates. However, the eight excavate taxonomic groups used for the EukRef databases (Table 1) are evident from the SSU rRNA phylogeny, albeit with the Jakobida, Euglenida and Glycomonada each appearing paraphyletic. From this, we could base the classification of SSU rRNA gene sequences within these groups, especially in order to propagate this classification to poorly classified environmental sequences.

**Table 1.** List of Excavata databases

Higher taxonomy	Database	# of SSU rRNA sequences	# of taxonomic ranks
**Eukaryota; Excavata; Metamonada**	Fornicata	103	8
**Eukaryota; Excavata; Metamonada**	Parabasalia	715	9
**Eukaryota; Excavata; Metamonada**	Preaxostyla	102	8
**Eukaryota; Excavata; Discoba**	Jakobida	114	8
**Eukaryota; Excavata; Discoba**	Heterolobosea	448	8
**Eukaryota; Excavata; Discoba; Euglenozoa**	Euglenida	856	10
**Eukaryota; Excavata; Discoba; Euglenozoa; Glycomonada**	Diplonemea	525	9
	Kinetoplastea	3258	11

### Excavate groups

#### Preaxostyla

The Preaxostyla database of SSU rRNA sequences is the smallest excavate database at 102 sequences (Figure S1). Eighty percent of these sequences are symbionts assigned to the Oxymonadida that reside in the hindguts of lower termites and *Cryptocercus* cockroaches and aid in the digestion of lignocellulose (22, 23, Figure 1C). The other 20% were derived from free-living organisms, usually from aquatic environments, and recently classified into two families, the Paratrimastigidae and Trimastigidae (24, Figures 1C and D). In the process of generating this database, three unclassified environmental sequences were assigned to the Preaxostyla—two of these were found from clay walls and assigned to the Paratrimastigidae, and one sequence from a marine cold seep, which did not cluster with any taxonomic group. Nucleic acids from oxymonads, with especially long and divergent SSU rRNA genes, are notoriously difficult to amplify (22, 25), which has likely negatively biased recovery of their sequences from environmental samples. Concerted efforts will be needed to discover further diversity from this group, particularly from anoxic environments.

As most of the preaxostylid data are from host-associated organisms, we have included host taxonomy in this database. However, the host classifications were taken directly from GenBank taxonomy databases and were not further curated. As EukRef expands, linking the taxonomy of symbionts with the curated taxonomy of their hosts will enable better investigations of host-symbiont evolution and diversification.

#### Fornicata

We have recovered 103 SSU rRNA sequences of Fornicata (Figure S2): of these, 74% were derived from isolated specimens (Figure 1B), with half (51% of total sequences) derived specifically from host-associated specimens (Figure 1C). We were able to improve the taxonomic classification of 95% of the environmental sequences using phylogenetics, as compared to their classification in GenBank. Based on current SSU rRNA diversity, Fornicata appears to be one of the smallest and least diverse groups within excavates. This might reflect the true diversity of Fornicata; however, many well-studied and morphologically described lineages of Fornicata are host-associated organisms with tremendously divergent rRNA sequences (diplomonads, retortamonads) (10, 11, 26–29). Therefore, the limited diversity of available Fornicata SSU rRNA sequences may be, at least partially, the result of primer bias or due to the lack of environmental DNA studies on the diversity of microbial eukaryotes in host-associated contexts.

#### Parabasalia

The vast majority (97%) of the 715 SSU rRNA sequences of Parabasalia are host-associated, and 74% are from known symbionts or parasites of animals (Figure 1B and C, Figure S3). Very few of these sequences originate from cultures, with 83% derived from cell isolations (Figure 1A). Only 11 sequences originated from free-living Parabasalia, but this is likely not a reflection of their true diversity.

The taxonomy of Parabasalia has recently been updated to reflect their molecular phylogeny (30). Based on this taxonomic revision, SSU rRNA sequences are classified into eight well-accepted major groups: Cristamonadida, Hypotrichomonadida, Spirotrichonymphida, Trichomonadida, Honigbergiellida, Tritrichomonadida, Trichonymphida and Lophomonadida. For Cristamonadida, mid-level taxonomic ranks are lacking to fully classify the SSU rRNA sequences largely because support for internal branches in molecular phylogenies is low and relationships among cristamonad taxa are not clear. As a result, all 91 Cristamonadida sequences were not classified with formally described mid-level taxonomic ranks, but instead, these ranks were filled with lower-level taxon names for sequences from organisms where this has been described (i.e. genus names). This was also the case for 54 parabasalid sequences from other major groups, mostly consisting of environmental clones that clustered independently of sequences having a complete classification. Molecular phylogenies continue to force revisions to parabasalid taxonomy, such as the placement of *Lophomonas* outside of the Cristamonadida despite sharing what were considered canonical morphological characters (31). With growing molecular data for parabasalid organisms coupled with a clearer understanding of morphological evolution, other major taxonomic revisions are likely, and updates to this database (and others) will be important.

#### Jakobida

The EukRef pipeline recovered 114 sequences reliably assigned to Jakobida (Figure S4): of these, 76% were derived from environmental sequencing studies, predominantly from low oxygen environments (Figure 1B). It is important to note that SSU rRNA-based phylogenetic trees generally do not recover a monophyletic Jakobida; rather the two subgroups, Andalucina and Histionina, form independent clades (13, 32, Figure 1A). However, monophyly of Jakobida is strongly supported by multigene phylogenies and by morphology (4, 32, 33). As such, this taxonomic group is largely accepted. Recently, Yabuki et al. (34) reported discovery of the heterotrophic nanoflagellate *Ophirina amphinema* and classified it as the third subgroup of jakobids (Ophirinina).

The Jakobida SSU rRNA gene sequences in our database represent six previously described genera, as well as ‘Seculamonas ecuadoriensis’ (*nomen nudum*, undescribed genus and species), five environmental clades, and four jakobid singleton sequences. All five identified environmental clades belong to the Andalucina: four clades are part of the taxon Stygiellidae and one is a sister lineage to the genus *Andalucia* (Andaluciidae). The four environmental clades of Stygiellidae were named Stygiellidae environmental clades I to IV (shortened as: STYG_1, STYG_2, STYG_3 and STYG_4), adopting the terminology introduced by Pánek *et al.* (13). The additional environmental clade was named AND_1.

#### Heterolobosea

Heterolobosea is a group of heterotrophic amoebae, flagellates and amoeboflagellates that can be sub-divided into two well-supported lineages, Pharyngomonada and Tetramitia (35, Figure 1A, Figure S5). The latter group is ecologically and morphologically extremely diverse and contains the vast majority of described species; however, its internal phylogeny is still almost exclusively based on SSU rRNA data. Because the traditional classification of Heterolobosea does not correspond with its internal phylogeny as it is currently understood, our database adopted provisional terminology as presented by Pánek *et al.* (36) and Hanousková *et al.* (37). In short, we recognize eight main clades of Tetramitia: Selenaionida (syn. Tetramitia clade VII), Acrasida (syn. Tetramitia clade II), Percolatea (syn. Tetramitia clade IV), Tetramitia clades I, III, V, VI and divergent family Creneidae that is known so far from a single culture (38).

The SSU rRNA gene is an excellent marker for the genus determination of Heterolobosea, but it provides only limited resolution at the species level in case of the genus *Naegleria* (39) that includes virtually one-third of the known Heterolobosea species diversity. In total, our database contains 448 sequences from Heterolobosea (Figure S5), with 155 of them assigned to the genus *Naegleria*. Another 65 sequences represent *Neovahlkampfia nana*. Although only 85 sequences come from environmental studies, we detected and named seven environmental clades. Three of them most likely represent undescribed deep lineages of Tetramitia (TET_VI_1, TET_VI_4 and PSA1), the other four branch close to existing genera: TET_VI_2 is sister to *Parafumarolamoeba*, TET_VI_3 to *Euplaesiobystra*, TET_VI_5 branches sister to *Vrihiamoeba*, and TET_I_1 is sister to the genus *Neovahlkampfia*. Three of these clades were detected exclusively in acidic environments (TET_VI_1, TET_VI_3 and PSA1).

#### Euglenozoa

The Euglenozoa were split into two databases, the Euglenida and the Glycomonada. The Glycomonada SSU rRNA gene sequences belonging to Kinetoplastea and Diplomonadea are described here separately due to their strongly supported monophyly and a differing number of taxonomic levels (Figure 1A, Table 1).

#### Euglenida

A total of 856 sequences belonging to Euglenida were recovered, all of them from nominally free-living organisms (Figure 1C, Figure S6 and Figure S7). Of these, 177 (almost all environmental—20.7%) branched with and were subsequently assigned to Symbiontida, whose position within Euglenozoa is still debated, but there is ultrastructural and modest molecular evidence that they fall within or sister to Euglenida (14). The Symbiontida were subdivided into Postgaardida, *Bihospites*, and clades SYMBT1 and SYMBT2. Only Postgaardida and *Bihospites* contain sequences identified to species, namely *Calkinsia aureus* and *Bihospites bacati*, with SYMBT1 and SYMBT2 as purely environmental clades. A total of 163 sequences including 111 environmental sequences fell among the clades of phagotrophic euglenids. Interestingly almost all of these environmental sequences (107, or 96%) were assigned to Petalomonadida, a clade of rigid cells that glide on their anterior flagellum. This bias toward Petalomonadida is likely due to the divergent nature of most other Euglenida SSU rRNA gene sequences, which makes it hard to capture this diversity using universal primers (40). Additionally, the V4 region of Euglenida tends to be massively expanded past the 600-bp limit of current high-throughput sequencing technology, but Petalomonadida represents an exception to this trend.

Altogether, 492 sequences were assigned to the photosynthetic Euglenida clade (Euglenophyceae) with only a tiny proportion (26 sequences, or 5%) originating from environmental sequences. This can again be explained by the divergent and highly variable SSU rRNA genes typical of these organisms (41). The majority of Euglenophyceae sequences are from freshwater, while few sequences come from soil and marine environments. This might reflect their natural distribution or point to an undersampling of diversity from soil and marine environments.

#### Diplonemea

Database searches yielded 525 SSU rRNA gene sequences inferred to belong to Diplonemea (Figure S8). They are a clade of heterotrophic flagellates overwhelmingly known from marine habitats. This previously homogenous group has now been sub-divided into four well-supported clades: Diplonemidae (14, 42), Hemistasiidae (14, 42, 43), Eupelagonemidae (44, 45) and the DSPD II clade (44). Their branching order cannot be reliably resolved on the basis of the SSU rRNA gene and the current taxon sampling. The largest fraction of recovered sequences belonged to hyperdiverse Eupelagonemidae (453 sequences, 86%). Of these, all but one, belonging to the uncultured type species *Eupelagonema oceanica* (45), lack any formal taxonomic description. An overwhelming majority of the Diplonemea sequences was environmental, with most obtained from marine plankton, some from a hydrothermal plume or oxygen-depleted sea water, while only six were benthic (Figures 1B and D). Only a handful of sequences (*Diplonema papillatum, Hemistasia phaeocysticola*, two strains of *Rhynchopus* and one sequence belonging to the Eupelagonemidae) were found to be likely host-associated. We kept the original taxonomic annotations for 12 sequences (2%) belonging to Diplonemidae and Hemistasiidae. We provide more detailed classification for 97% of the sequences, most of which were previously annotated as ‘uncultured eukaryotes’ or ‘uncultured diplonemids’, and three erroneous annotations have been corrected. It is predicted that a vast undiscovered diversity is hidden within the taxon Eupelagonemidae (17).

#### Kinetoplastea

The Kinetoplastea comprises the largest number of excavate SSU rRNA sequences. Kinetoplastea can be sub-divided into three highly supported clades/subclasses (46, Figure S9: Metakinetoplastina (2947 sequences), Prokinetoplastina (295 sequences) and KIN1 (= Kinetoplastea clade 1) branching sister to the rest of the group. KIN1 comprises 16 environmental sequences from various deep-marine environments. For Prokinetoplastina, four clades are well-supported: *Ichthyobodo* (a parasite of fish), *Perkinsela* (an obligate endosymbiont of amoebae), and two aquatic environmental lineages of which one is comprised of sequences found exclusively in marine sediments, leaving 53% of sequences unclassified within the Prokinetoplastina. Metakinetoplastina contain four subgroups (Neobodonida, Parabodonida, Eubodonida, and Trypanosomatida), with the Neobodonida being the deepest branching clade, and the Trypanosomatida being most closely related to the Eubodonida (46–49). In our Kinetoplastea tree, Parabodonida are strongly supported (95% bootstrap support), Trypanosomatida moderately supported (79%), Eubodonida poorly supported (30%), and Neobodonida (1182 sequences) is not monophyletic, but instead represented by a paraphyletic cluster of clades at the base of Metakinetoplastina. Some of these clades (especially *Dimastigella* spp., *Neobodo* spp., *Rhynchomonas* spp., and the clade MET7) seem to be important in marine environments (50). Taking into account the topology of our tree and previous publications (14, 46, 49, 51–56), we cannot rule out the possibility that Neobodonida are paraphyletic, and we decided to omit the term Neobodonida from our classification and classify their sequences as Metakinetoplastina.

Parabodonida contains four clades corresponding to four described genera: *Parabodo* (free-living organisms from terrestrial and freshwater biomes and potential parasites found in plant sap), *Cryptobia* (endoparasites of snails), *Procryptobia* (free-living marine organisms), and *Trypanoplasma* (fish blood parasites; including some species originally assigned to *Cryptobia*). Eubodonida are free-living bacterivorous protists found in soil, freshwater and marine habitats. Most of their sequences were originally annotated as uncultured bodonids, uncultured eukaryotes or *Bodo saltans—*a genetically diverse morphospecies likely representing multiple species (46, 53). In Metakinetoplastina, most of the sampling and sequencing efforts have been dedicated to parasitic Trypanosomatida, and the level of misannotations was generally very low. Comprising 1633 SSU rRNA gene sequences, Trypanosomatida are the most abundant taxon of excavates in our databases, with 1138 (70%) of those sequences belonging to the genus *Trypanosoma*.

Ninety-four percent of Kinetoplastea sequences were correctly annotated to seven taxonomic ranks, and 6% (208 sequences) were poorly annotated as ‘uncultured eukaryote’ or ‘uncultured euglenozoan’. We kept the original taxonomic annotations for 50% (or 1626) of Kinetoplastea sequences, provided a more detailed classification for 46% (or 1507) of the sequences, and corrected 4% (or 125) classifications. Nine well-supported Kinetoplastea clades do not have any described representatives and are known only from environmental sequences: the KIN1 clade, two Prokinetoplastina clades (PRO1 and PRO2), and six basal Metakinetoplastina clades (MET1, MET2, MET4, MET5, MET7 and MET9).

## Concluding remarks

Overall, we have recovered 6121 sequences from the NCBI NR database that can be reliably assigned to the excavate groups Metamonada and Discoba (Table 1), summarizing our current understanding of the diversity of excavates based on SSU rRNA data. We have improved on the GenBank classification and corrected the assignment of many sequences such that all of the sequences now have rational higher-level taxonomic labels. A majority of described Metamonada and Discoba species are characterized exclusively by morphological features with no molecular data available; consequently, we have a limited ability to assign species labels to the molecular diversity of Excavata in environmental samples. On the other hand, careful analyses of molecular data from environmental studies based on our database should help significantly to reveal the true diversity of existing excavate groups: for instance, analyses using our database revealed novel diversity within Diplonemea and Kinetoplastea (17, 50).

The presented EukRef databases highlight several interesting patterns in the diversity of excavates and exposes the uneven and varied historical effort of protistologists in the study of these groups. First of all, for several groups, there is a large imbalance between environment-, culture-, or isolate-derived data (Figure 1B). Most of the Heterolobosea, Fornicata, and Parabasalia sequences come from uncultured isolates, while jakobids and diplonemids are mostly derived from environmental sequences, but most Euglenida come from established cultures in culture collections. Diplonemea represent an extreme case of a highly diverse group (17), with only a few cultured species (42, 43, Figure 1B). These differences likely represent biases caused by the fact that representatives of some groups are generally easier to cultivate than others. The databases are also likely biased toward taxa that predominantly live in easily accessible environments (e.g. freshwater ponds) or from geographical locations with many active protistologists (e.g. North America and Europe). North America and Europe are the most commonly sampled locations, while Africa, the Arctic and Antarctica are the least sampled locations, which suggests that tropical and polar biodiversity is poorly investigated (Figure 1E). There is also an apparent lack of environmental studies aimed at predominantly host-associated groups such as Fornicata, Preaxostyla or Parabasalia (Figures 1C and D). This is particularly disconcerting as a large part of the known diversity of Preaxostyla and Parabasalia is described from terrestrial animals, and these protists are typically transferred directly from host-to-host without having a free-living or cyst stage (as opposed to many parasites of aquatic organisms). Consequently, a significant part of the diversity of these clades will be excluded from most environmental studies, along with the hosts. The current trend in sequencing short amplicons encourages analyses of SSU rRNA from host environments, which can reveal novel diversity; however, due to the divergence of SSU rRNA in many eukaryotes, unknown groups may be missed and longer sequences are necessary to more fully resolve the taxonomic and phylogenetic affinities of eukaryotes.

The number of curated SSU rRNA gene sequences, as obtained through the EukRef pipeline, varied for each group of interest. For example, the Glycomonada (Diplonemea + Kinetoplastea) database includes by far the most sequences (n = 3783, 62% of all recovered sequences, Table 1), while Preaxostyla (n = 102) and Fornicata (n = 104) are the smallest groups (Table 1). These disparities in diversity could accurately represent the extant diversity. However, as discussed above, these differences are more likely caused by experimental and sampling biases, therefore much more diversity is awaiting discovery. The high genetic divergence of the SSU rRNA gene might be partially responsible for this bias and proper investigation of the diversity of some of the excavate groups might require using group-specific primers and longer sequences than the usual short sequence tags.

## Experimental procedures

### Generation of the SSU rRNA sequence databases

Following the EukRef pipeline (eukref.org), seven datasets of SSU rRNA sequences were generated, each representing the breadth of diversity within five major taxa of the Excavata (Fornicata, Parabasalia, Preaxostyla, Jakobida and Heterolobosea), and two separate groups within a sixth major taxon, Euglenozoa (Euglenida and Glycomonada). Iterated BLASTN searches were used to retrieve all SSU rRNA sequences deposited in GenBank (www.ncbi.nlm.nih.gov) and Silva r128 (www.arb-silva.de) with at least 70% similarity to the sequences in each dataset. Sequences identified as chimeras and short sequences (>500 bp) were removed from each database. Sequences were clustered using a threshold of 97% similarity, and the centroid sequence of each cluster was used to build a phylogenetic tree, except for Euglenozoa where a phylogenetic tree was built from all sequences without clustering. For each dataset, several distantly related SSU rRNA gene sequences were included as outgroups to root the phylogenetic tree. Phylogenetic trees were constructed following the EukRef pipeline (alignments using MAFFT, trimming using trimAl, building trees using RAxML with the GTRCAT model and 100 bootstrap replicates). The phylogenetic tree for each dataset was visually inspected, and sequences resulting in long, errant branches were removed from the dataset. A final SSU rRNA reference tree was generated for each curated sequence dataset (Figures S1–S9).

### Assigning taxonomy to SSU rRNA sequences

Using the SSU rRNA reference trees, a full classification was manually assigned to each tip based on phylogenetic support and currently accepted taxonomy. Classification was assigned for each taxonomic level for each sequence based on the position of each sequence in a phylogenetic clade. If necessary, the taxonomy for a sequence was modified to be consistent with all other sequences in a clade following the best studied or type species. Empty taxonomic ranks were labeled with the taxon name of the higher rank. Well-supported clades consisting of unclassified environmental sequences were given abbreviated names (a prefix) of the lowest assigned taxon in capital letters followed by a number according to EukRef guidelines. The classification entered for the sequences is based on the phylogenetic resolution provided by the SSU rRNA trees. A taxon name was not entered for a taxonomic level in the database if it was not possible to distinguish sequences associated with this taxon name as a distinct clade from the SSU rRNA trees, particularly at the species level (even though sequences may be derived from organisms described as different species, or even deeper taxa, based on other data). For these cases, these taxonomic levels were filled with the taxon name at the higher level. The classification given to each tip in the tree, based on the phylogeny of the centroid sequence from a 97% similarity cluster, was propagated to all sequences in the cluster (with the exception of Euglenozoa that were not clustered due to loss of resolution, and classification was assessed for every sequence). This means that all sequences in a 97% similarity cluster are classified consistently reflecting the taxonomic limitations inherent in SSU rRNA data. Metadata for each sequence was obtained from GenBank entries or by referring to research publications and culture collection databases. Descriptions of environmental biomes and environmental materials used standardized terms from http://www.environmentontology.org/Browse-EnvO.

### Excavate phylogenetic tree construction

All excavate SSU rRNA sequences representing 97% similarity clusters and greater than 1000 bp in length (1678 sequences) were combined for a single phylogenetic analysis along with *Malawimonas jakobiformis* (AY117420), *Tsukubamonas globosa* (AB576851), and 19 outgroup sequences from diverse other eukaryotes including *Cyanophora paradoxa* (AY823716), *Glaucocystis nostochinearum* (X70803), *Leptomyxa reticulata* (AF293898), *Acanthamoeba castellanii* (KF318462), *Ustilago maydis* (X62396), *Neurospora crassa* (FJ610444), *Saccharomyces cerevisiae* (AAEG01000105), *Glomus* sp. (AJ852533), *Allomyces arbuscula* (NG_017166), *Monosiga brevicollis* (AF084618), *Proterospongia choanojuncta* (AY149896), *Trichoplax adhaerens* (AY652578), *Oryza sativa* (AF069218), *Arabidopsis thaliana* (X16077), *Physcomitrella patens* (X80986), *Micromonas pusilla* (AB183589), *Polytomella parva* (D86497), *Chlamydomo-nas reinhardtii* (AB511834) and *Volvox carteri* (X53904). The SSU rRNA gene sequences were aligned using MAFFT and trimmed using trimAl, for an analysis of 926 sites (57–59). Maximum likelihood was used to construct a phylogenetic tree using a GTRCAT model of nucleotide substitution implemented in RAxML (60). Statistical support for each branch was calculated based on 1000 bootstrap replicates.

## Supplementary Material

baaa080_Supp
