# Supplementary material for: EukRef-excavates: seven curated SSU ribosomal RNA gene databases
Source: Database (Oxford). 2020 Nov 20;2020:baaa080. doi: 10.1093/database/baaa080 (PMC7678783; doi:10.1093/database/baaa080)

Figures S1 – S9. Maximum likelihood phylogenetic trees for each specific EukRef database constructed in RAxML using GTRCAT model. Statistical support is derived from 100 bootstrap replicates. Euglenida and Glycomonada datasets were each split into two separate tree figures as they both contain very large number of sequences. Trees are outgroup rooted. Tip labels are the final classification in the databases.

Figure S1
Preaxostyla

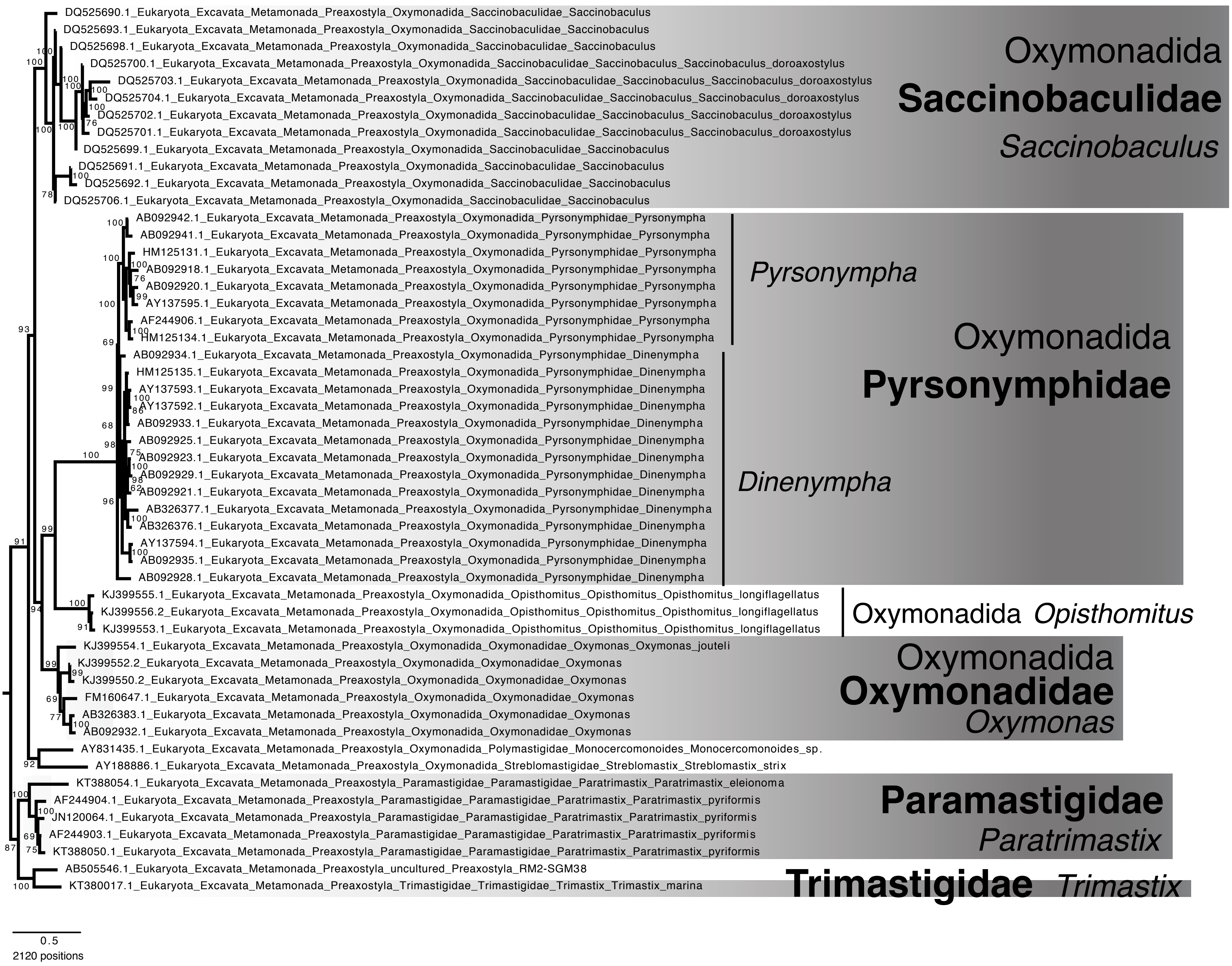

Figure S2
Fornicata

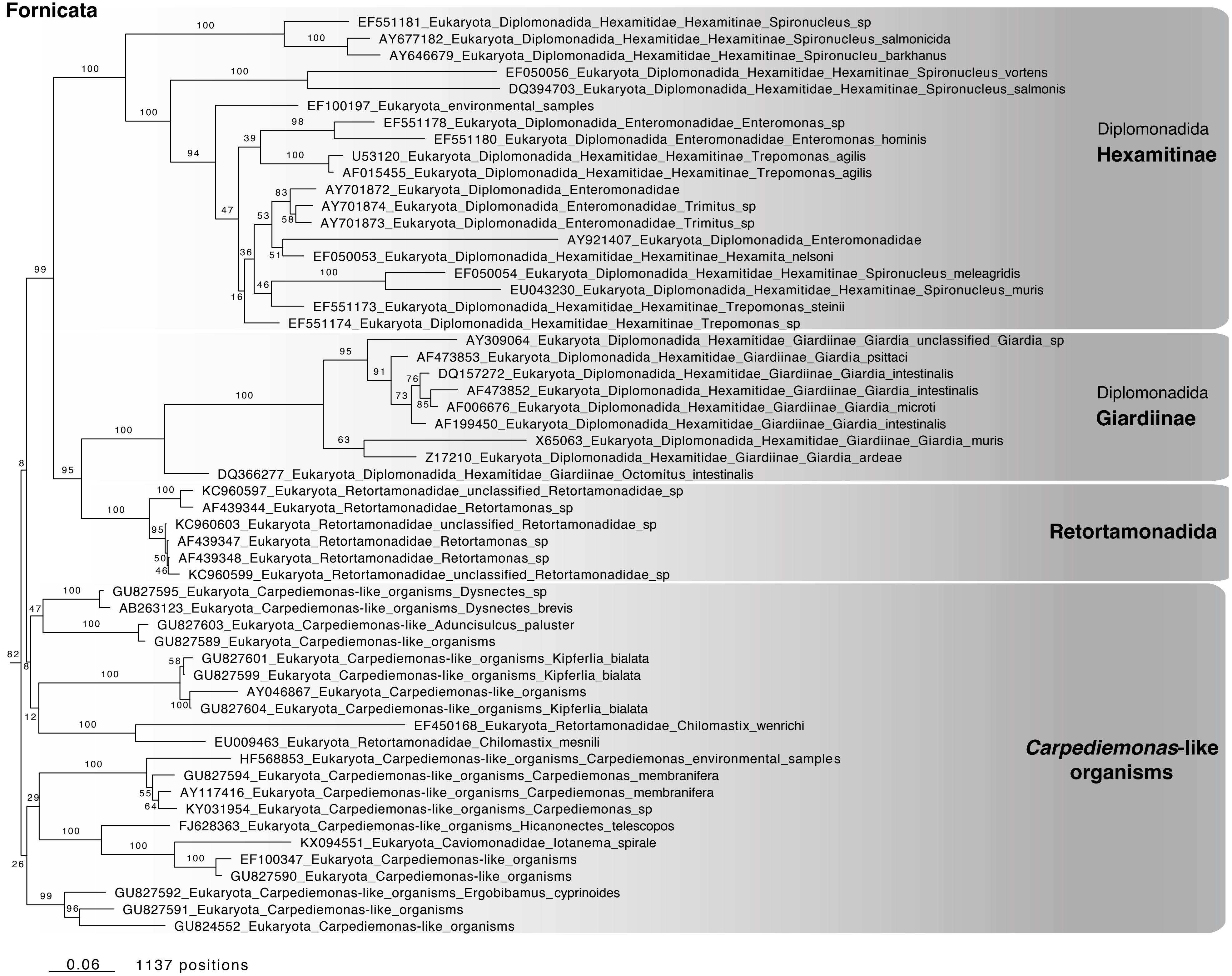

Figure S3
Parabasalia

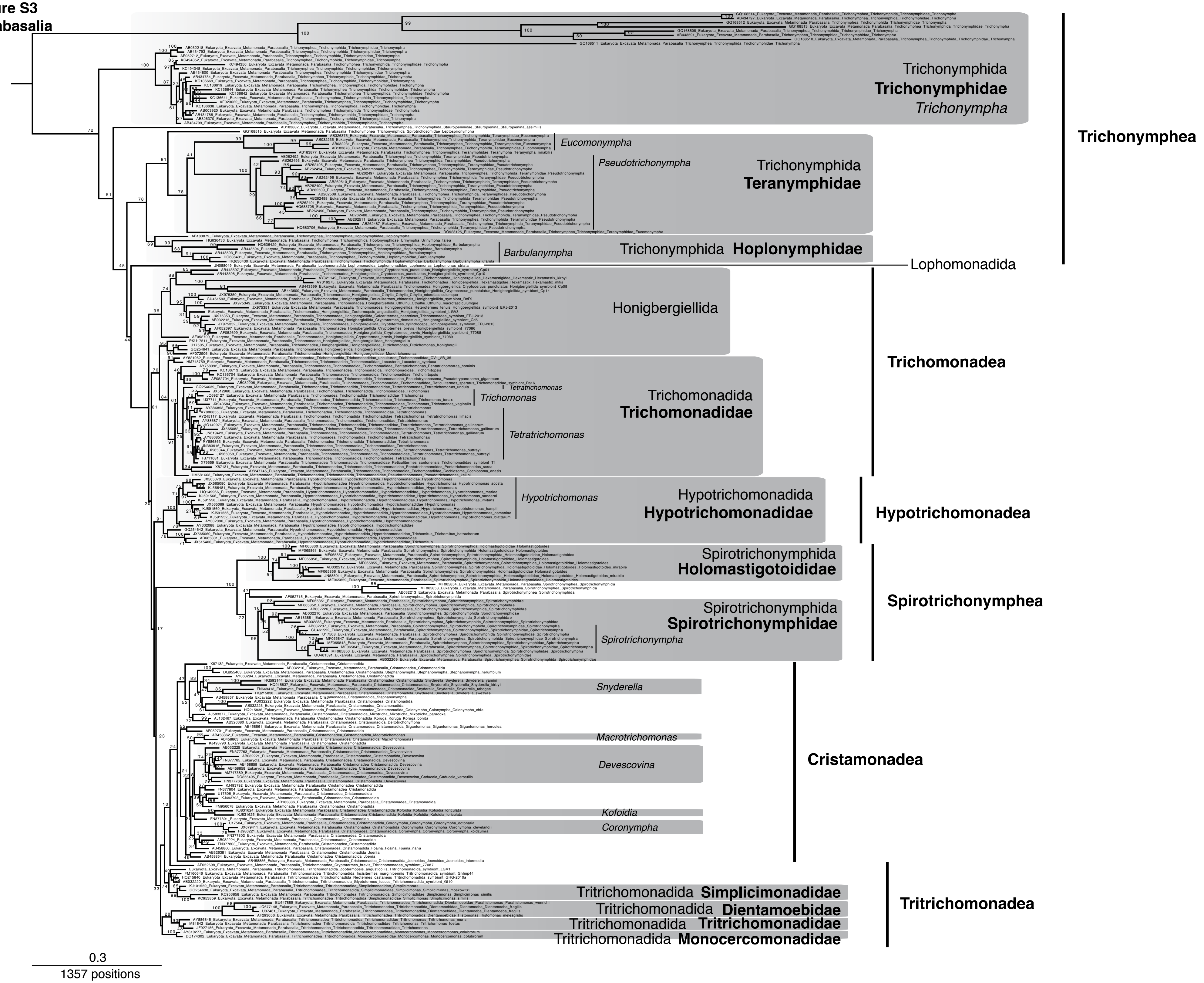

Figure S4
Jakobida

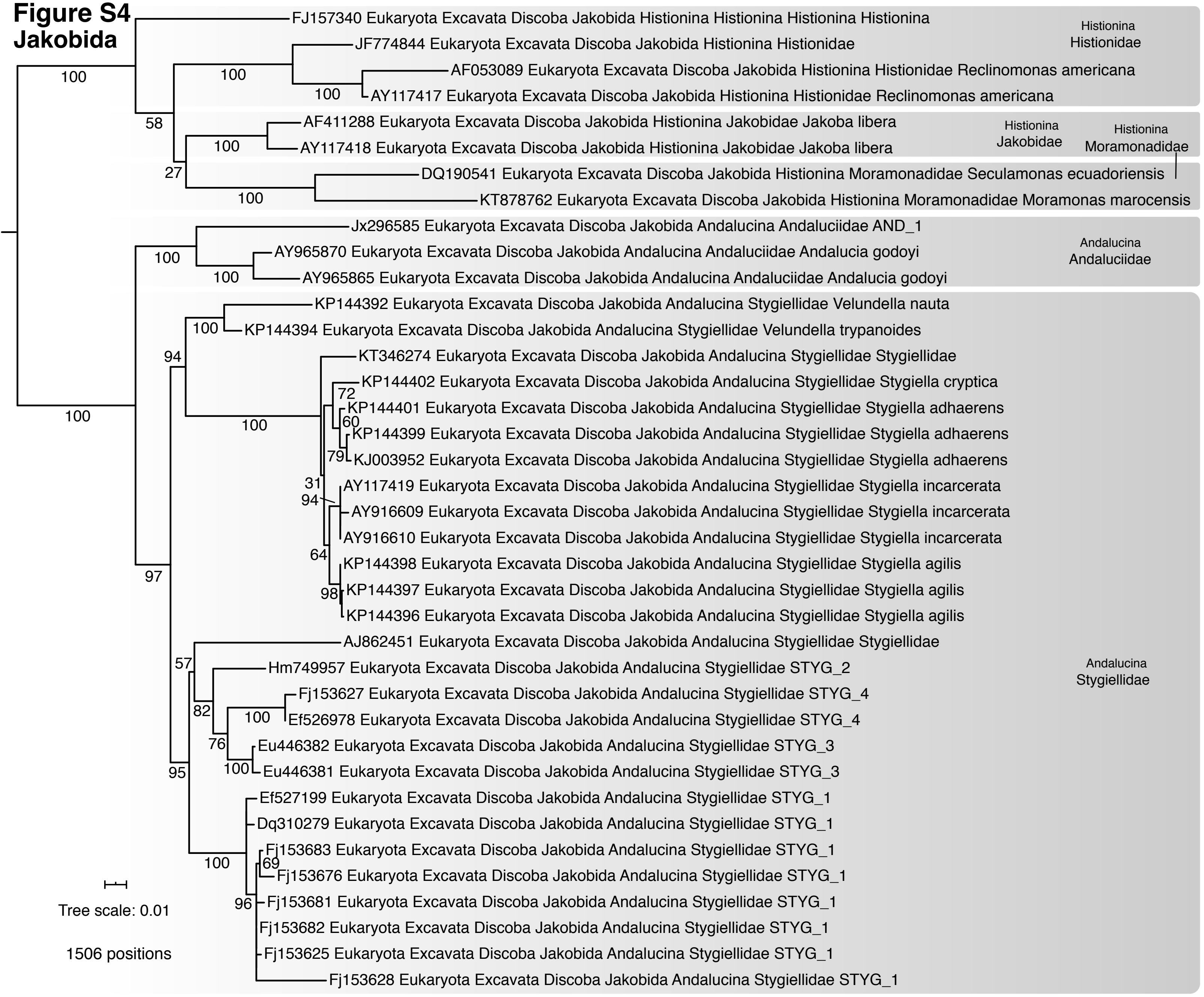

Figure S5
Heterolobosea

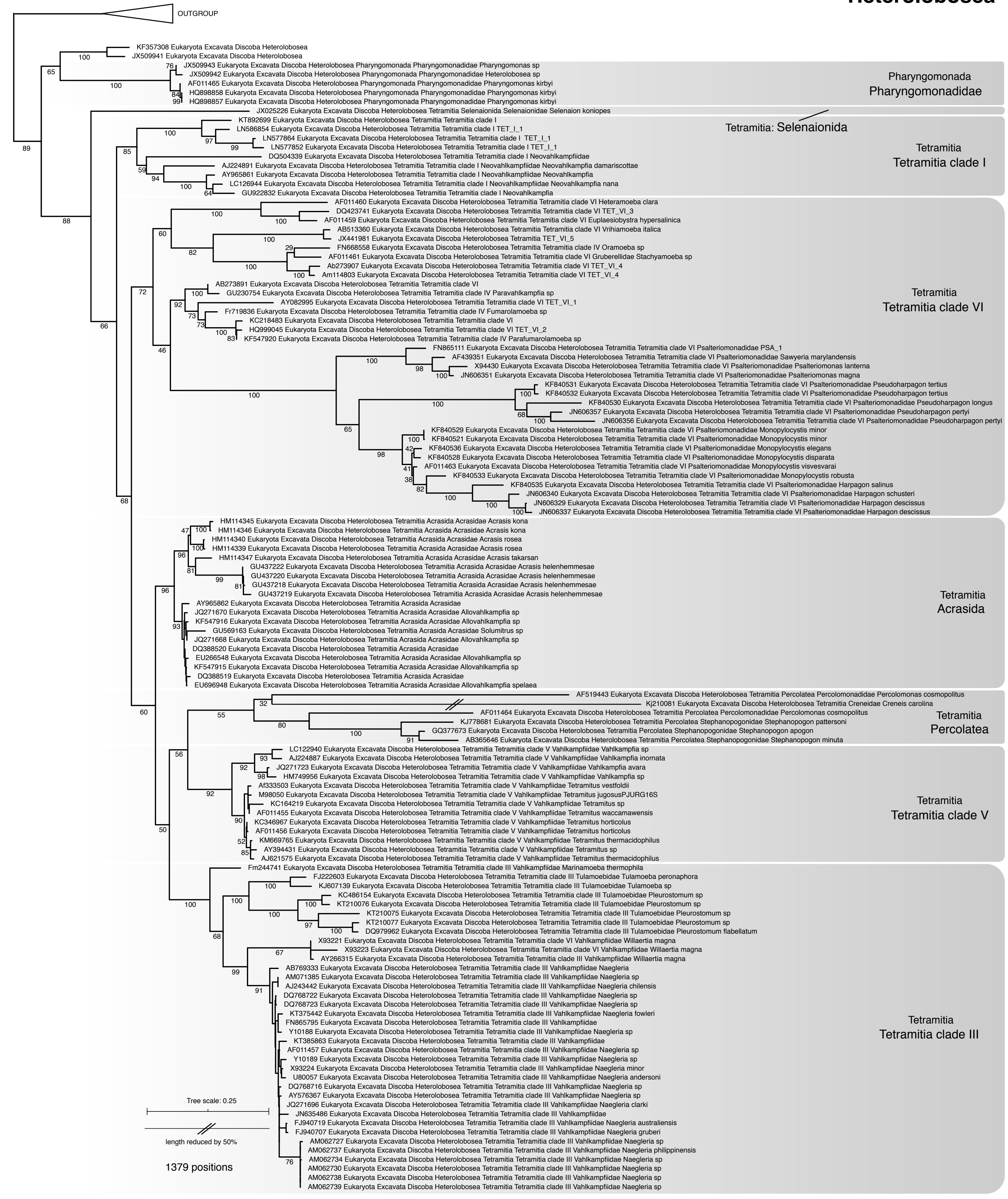

Figure S6
Euglenida 1

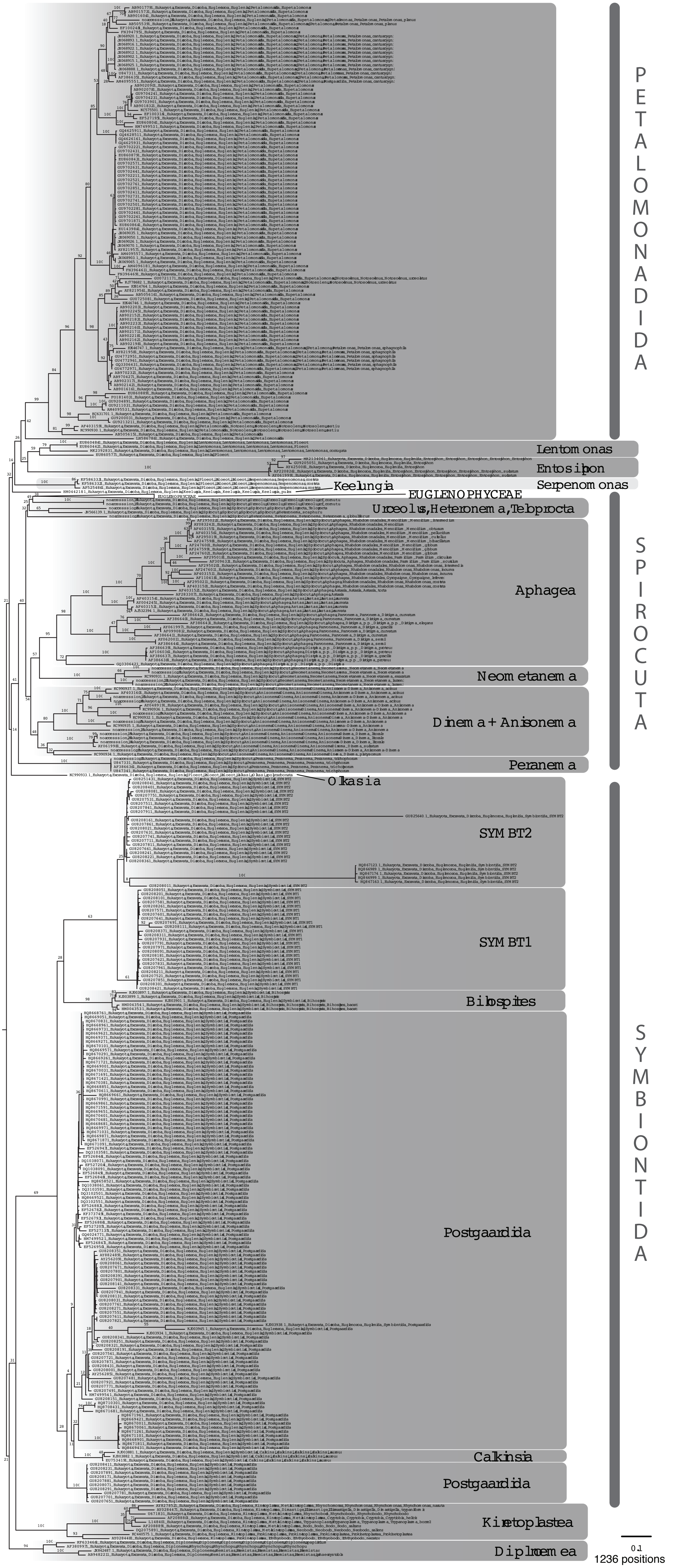

Figure S7
Euglenida 2

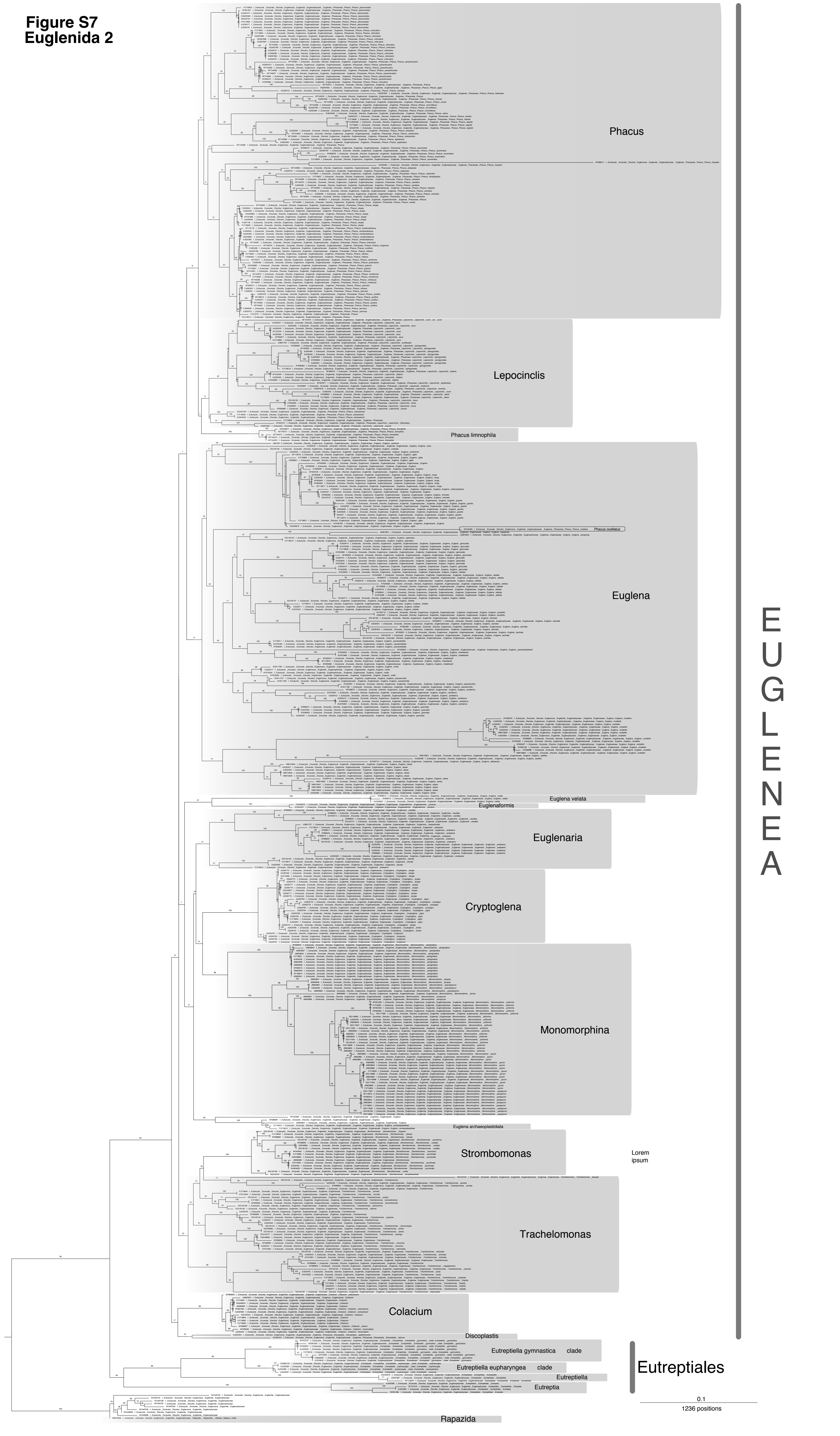

Figure S8

Diplonemea

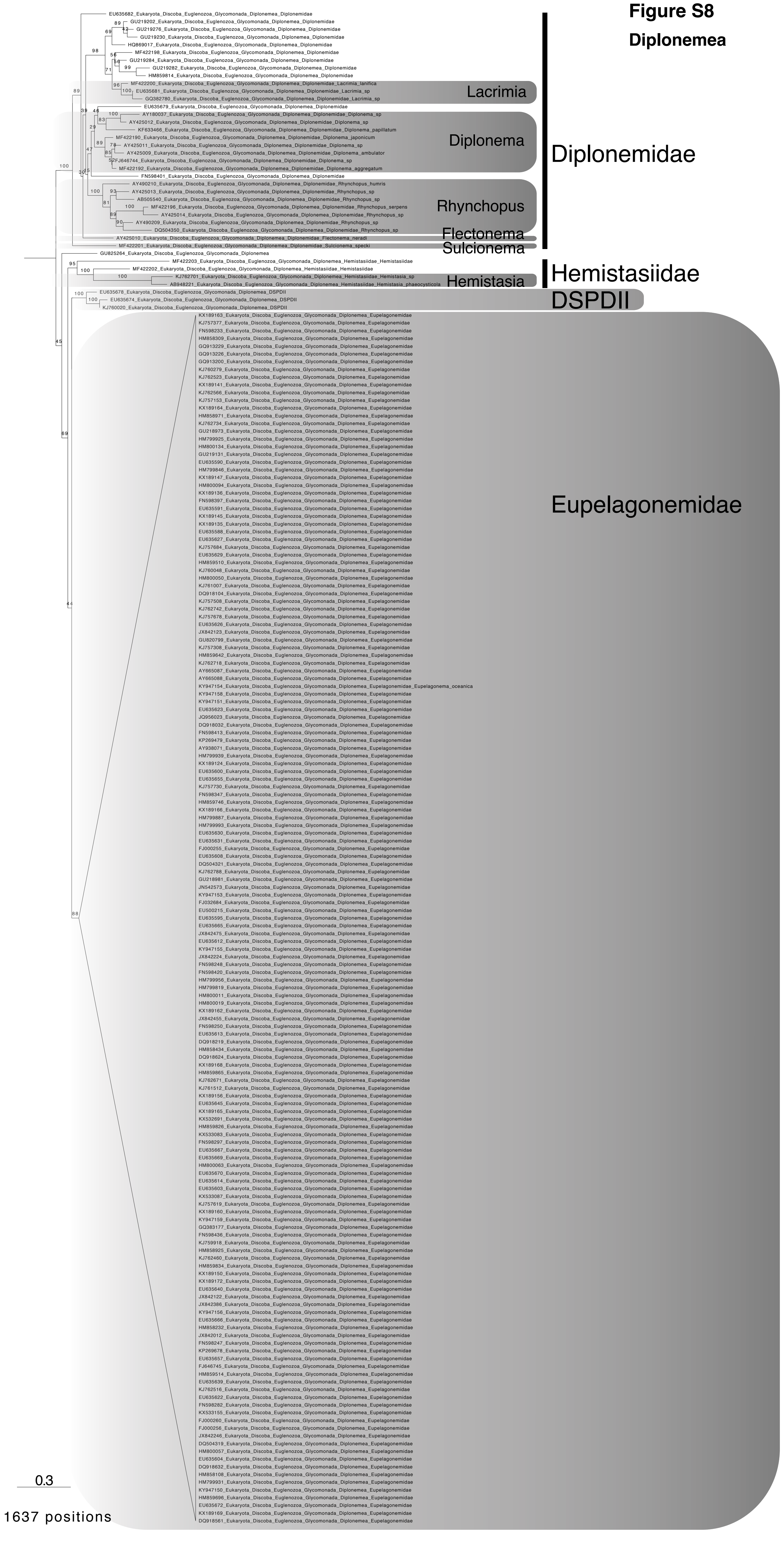

Figure S9
Kinetoplastea

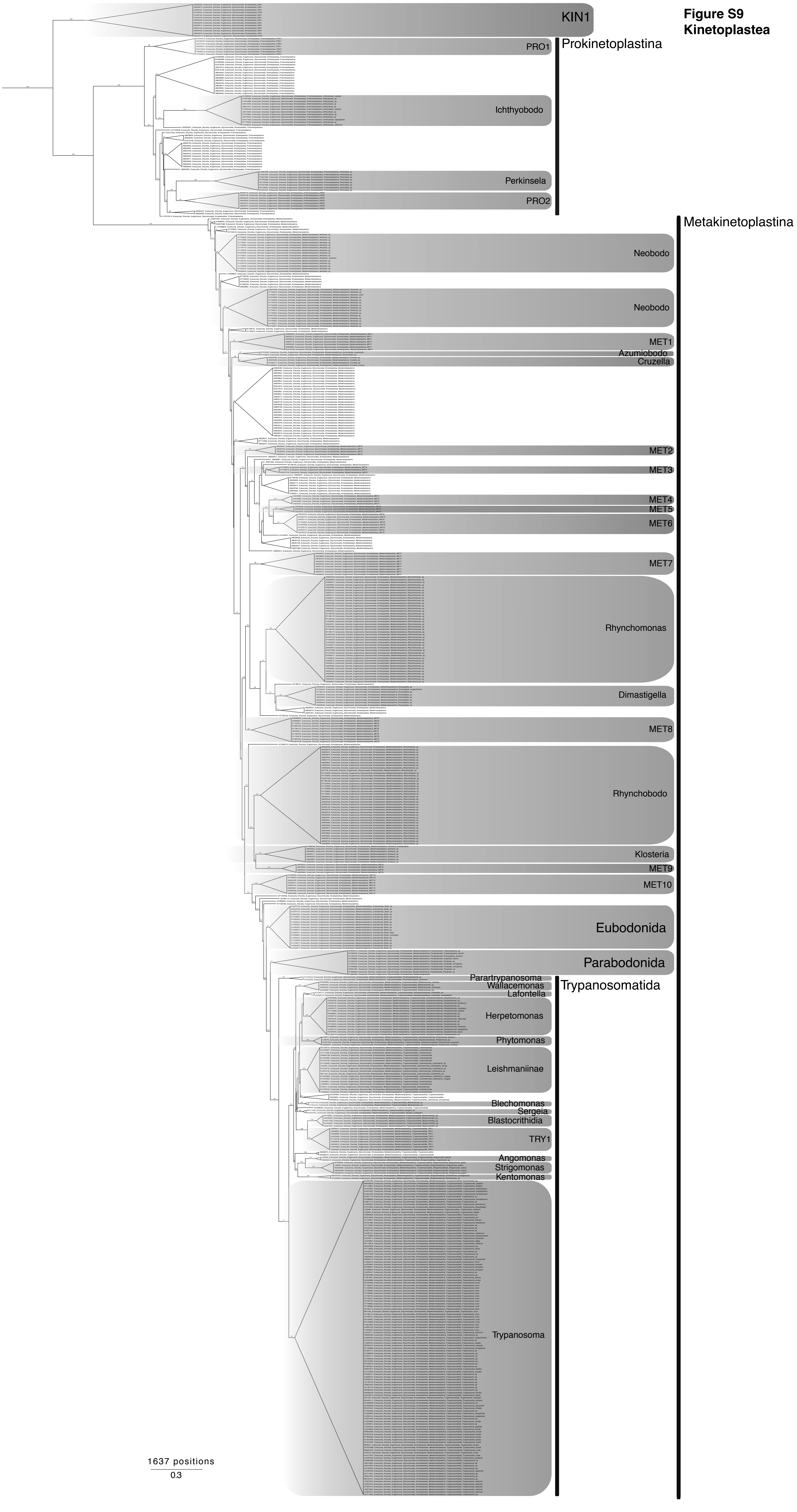

Supplement: baaa080_Supp [file baaa080_supp.zip › Supplementary_Materials_Revised.pdf]
